# Evaluation of a biomarker for amyotrophic lateral sclerosis derived from a hypomethylated DNA signature of human motor neurons

**DOI:** 10.21203/rs.3.rs-5397445/v1

**Published:** 2024-11-26

**Authors:** Calum Harvey, Alicja Nowak, Sai Zhang, Tobias Moll, Annika K Weimer, Aina Mogas Barcons, Cleide Dos Santos Souza, Laura Ferraiuolo, Kevin Kenna, Noah Zaitlen, Christa Caggiano, Pamela J Shaw, Michael P Snyder, Jonathan Mill, Eilis Hannon, Johnathan Cooper-Knock

**Affiliations:** University of Sheffield; University of Sheffield; University of Florida; University of Sheffield; Novo Nordisk Foundation Center for Genomic Mechanisms of Disease, Broad Institute; University of Sheffield; University of Sheffield; University of Sheffield; University Medical Center Utrecht; UCLA; UCLA; University of Sheffield; Stanford University School of Medicine; University of Exeter Medical School, University of Exeter; University of Exeter Medical School, University of Exeter; University of Sheffield

## Abstract

Amyotrophic lateral sclerosis (ALS) lacks a specific biomarker, but is defined by relatively selective toxicity to motor neurons (MN). As others have highlighted, this offers an opportunity to develop a sensitive and specific biomarker based on detection of DNA released from dying MN within accessible biofluids. Here we have performed whole genome bisulfite sequencing (WGBS) of iPSC-derived MN from neurologically normal individuals. By comparing MN methylation with an atlas of tissue methylation we have derived a MN-specific signature of hypomethylated genomic regions, which accords with genes important for MN function. Through simulation we have optimised the selection of regions for biomarker detection in plasma and CSF cell-free DNA (cfDNA). However, we show that MN-derived DNA is not detectable via WGBS in plasma cfDNA. In support of our experimental finding, we show theoretically that the relative sparsity of lower MN sets a limit on the proportion of plasma cfDNA derived from MN which is below the threshold for detection of WGBS. Our findings are important for the ongoing development of ALS biomarkers. The MN-specific hypomethylated genomic regions we have derived could be usefully combined with more sensitive detection methods and perhaps with study of CSF instead of plasma. Indeed we demonstrate that neuronal-derived DNA is detectable in CSF. Our work is relevant for all diseases featuring death of rare cell-types.

## Introduction

Amyotrophic lateral sclerosis (ALS) is an incurable neurodegenerative disease where death results from motor neuron (MN) loss leading to respiratory failure. The design and development of novel therapeutics has been held back because of the lack of a specific biomarker. Currently, neurofilament proteins measured in plasma provide a non-specific readout of neuronal death [1]. Neurofilament proteins form important structural components of the large myelinated axons which are found in MN. MN death triggers the release of neurofilaments from the cytoplasm into the extracellular space [2]; as a result the level of detectable neurofilament is a function of the rate of MN death, and thus neurofilament measurement can be used as a biomarker of disease progression [1]. However, neurofilaments are not specific to MN and it is notable that serum neurofilament light chain (NfL) [3] is elevated in other neurological diseases. Indeed, for diagnosis of ALS, serum NfL is of limited value [4] even if it is useful for measuring the rate of progression. It follows that detection of a different marker which is released *only* from dying MN may outperform neurofilaments as a biomarker for ALS.

DNA methylation is fundamental to the control of gene expression and by inference, genomic methylation should be relatively cell specific. Cell-specific DNA methylation signals are stable between individuals, as was confirmed by a recent atlas of DNA methylation [5]. Moreover, DNA methylation is relatively stable over time [6]. Cell-free DNA (cfDNA) found in peripheral blood is the product of release from dying cells [7] and has been extensively proposed as a source of biomarkers in the cancer field [8]; methylated cfDNA is now the basis of FDA-approved applications e.g. [9]. We hypothesised that a DNA methylation signature which is specific to MN, and is detectable within cfDNA, might be both sensitive and specific as a biomarker of the rate of MN death due to ALS.

**Figure 1:**
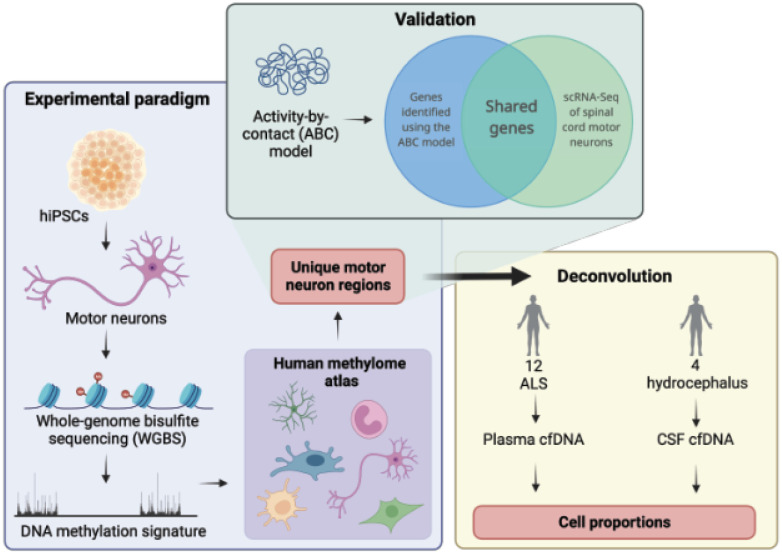
Derivation and biomarker evaluation of a hypomethylated DNA signature from whole genome bisulfite sequencing (WGBS) of human motor neurons. MN-specific DNA hypomethylation was used to assess the proportion of MN DNA within cfDNA in plasma from ALS patients (n=12) and CSF from controls (n=4). We sort to verify the validity of MN-specific DNA hypomethylated regions by linking regions to target genes and cross-checking those genes with independent observations of MN gene expression; we hypothesised that correctly identified hypomethylated regions should indicate regions of open, active and transcribed chromatin which should be statistically enriched in measures of MN-specific gene expression. We linked regions to target genes using the activity-by-contact (ABC) model [13].

We present whole genome bisulfite sequencing (WGBS) data from iPSC-derived MN from controls. These data complement our previously published epigenetic profiling from the same neurons [10]. It is practically difficult to obtain MN in sufficient quantity from post-mortem material to perform WGBS and therefore we chose to focus on iPSC-derived MN which are a gold-standard model of ALS [11]. We have published WGBS of cfDNA from ALS patients and controls [12] but previously we lacked a MN signature for comparison. Here we show, using simulation and measurement, that MN-specific DNA methylation is not detectable within cfDNA in plasma by WGBS. Future work will evaluate our MN DNA methylation signature by other means and in other biofluids. Our approach is summarised in Fig. 1.

## Results

### Cell-specific DNA methylation within control iPSC-derived MN is similar to human adult CNS neurons

WGBS was performed at high depth to profile DNA methylation within iPSC-derived MN from three neurologically normal individuals (Supplementary Table 1, Methods). A first question was whether the methylation signature of these neurons, which are derived *in vitro*, is consistent with CNS neurons abstained from human tissue.

**Figure 2:**
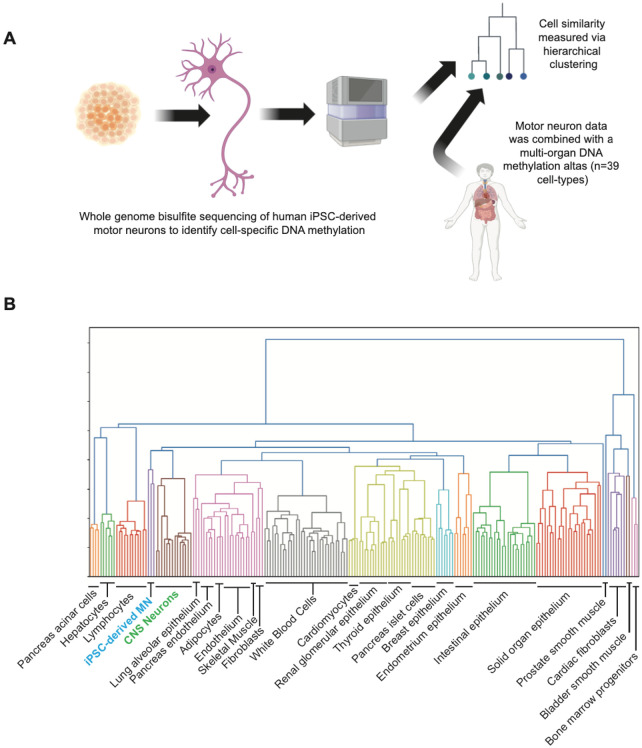
iPSC-derived MN maintain a DNA methylation signature consistent with human adult neurons. (**A**) Whole genome bisulfite sequencing (WGBS) of genomic DNA derived from human iPSC-derived MN was used to derive a profile of genomic methylation within MN for comparison with methylation profiles of 205 samples covering 39 cell-types [5]. (**B**) Unsupervised clustering was used to assess cell-similarity and revealed that iPSC-derived MN (blue text) cluster together with human CNS neurons (green text).

WGBS sequencing data were processed and quality control (QC) was performed according to the ENCODE 4 standards [14]. Methylation profiles of 205 samples covering 39 cell-types from an available methylation atlas [5] were combined with our samples, then used to segment the genome into blocks of co-methylated CpGs (Methods). Hierarchical unsupervised clustering was used to examine the relationships between samples (Methods, Fig. 2A). As expected, genome methylation within iPSC-derived MN clustered closely with CNS neuronal subtypes (Fig. 2B). On this basis we proceeded to use our data to identify MN-specific methylation (Methods).

### Identification of cell-specific hypomethylated genomic regions

Next we derived DNA methylation changes specific to MN via comparison with the methylation profiles of 205 samples covering 39 cell-types from an available methylation atlas [5]. Blocks of co-methylated CpGs that exhibited hyper- or hypomethylation specifically in MN were identified (Methods) and taken forward for further analysis. In total 8,729 regions were specifically hypomethylated in MN (Supplementary Table 2); hypomethylation indicates increased genomic accessibility suggestive of MN-specific function. A similar analysis identified 5,690 blocks which were specifically hypomethylated in the total set of human CNS neurons compared to other cell-types. The number of regions identified per cell-type varied dramatically from 61,693 for gallbladder to 436 for colon fibroblasts.

### MN-specific DNA methylation is linked to MN function but not to genetic risk for ALS

**Figure 3:**
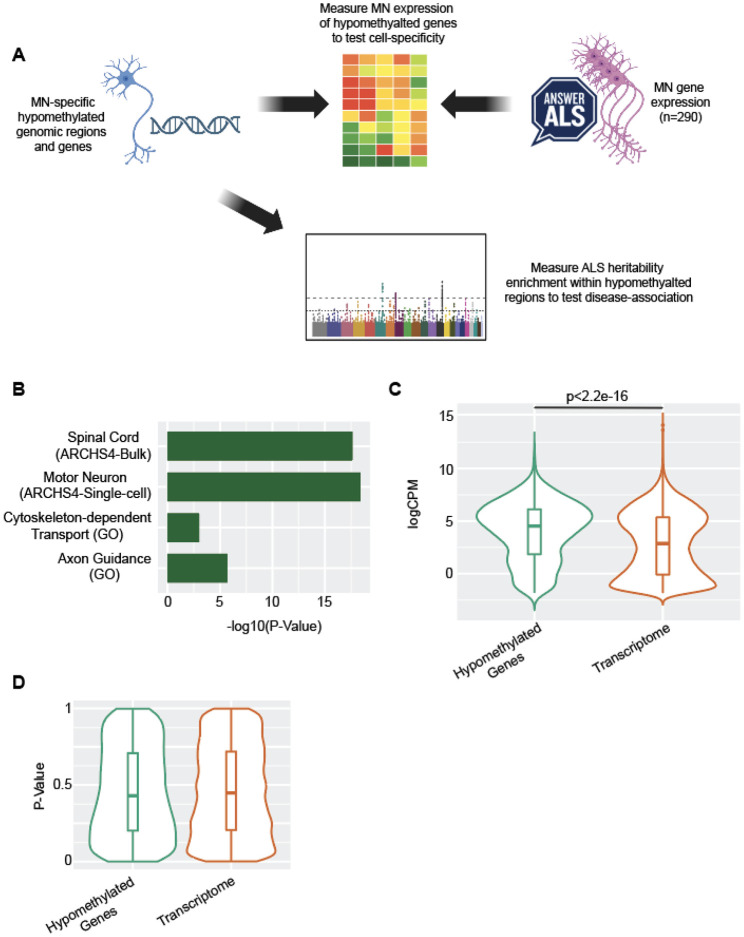
MN specific DNA methylation is linked to MN function but not to genetic risk for ALS. (**A**) We used independent measurements of MN gene expression and ALS heritability to verify the biological validity of identified MN-specific hypomethylated genomic regions. MN-specific hypomethylated genes are enriched with genes expressed in human MN (**B**) and in human iPSC-derived MN (**C**). MN-specific hypomethylated genes are not differentially expressed in ALS iPSC-derived MN compared to control MN (**D**).

Cell-specific DNA methylation is typically hypomethylated [5], which should be coincident with increased accessibility of underlying DNA over regulatory regions including enhancers [15]. As a validation of the regions we have identified, we examined the overlap of MN-specific hypomethylated enhancers and their target genes, with independent measurements of MN gene expression and ALS heritability (Fig. 3A).

To derive associated genes from MN-specific hypomethylated DNA blocks, we applied the activity-by-contact (ABC) model [13] to link regulatory regions to expressed genes within iPSC-derived MN (Methods). We found the total list of hypomethylated regions is associated with 2,046 expressed genes. We then tested this gene list for enrichment with human cell types and tissues included in ARCHS4 [16] using Enrichr [17], and found they were most significantly enriched for genes expressed specifically in spinal motor neurons isolated from post-mortem tissue [18] (Fisher’s exact test, p = 4.22e-19, OR = 1.79, using the ARCHS4 database [16], Fig. 3B). This demonstrates that the methylation profiles of the iPSC derived motor neurons are congruent with transcriptional profiles of human motor neurons.

To further characterise the function of MN-specific hypomethylated genes we examined RNA-sequencing from iPSC-derived motor neurons obtained from 245 ALS patients and 45 controls (www.answerals.org) (Methods). Genes linked to hypomethylated regions in MN were highly expressed within iPSC-derived MN compared to the background transcriptome (Wilcox rank sum test, p < 2.2e-16, Fig. 3C) which is consistent with an important role in MN function. Four genes were reported as differentially expressed (FDR < 0.05, negative binomial test) between ALS patients and controls in this data, but genes linked to hypomethylated regions in MN were not enriched within ALS-associated differentially expressed genes (Wilcoxon rank sum test, p = 0.25, Fig. 3D).

Finally, we performed LDSC [19] using a recent GWAS study of ALS [20] to examine disease-specific heritability enrichment within MN-specific hypomethylated regions. Heritability for ALS was enriched within hypomethylated regions but this was not statistically significant (OR = 25.2, se = 26.05, p = 0.38, LDSC, Methods). We conclude that MN-specific DNA hypomethylation is associated with gene expression linked to MN function, but we find no conclusive evidence that there is a specific association with genes dysregulated in MN in a disease context.

### An optimum set of hypomethylated DNA regions for ALS biomarker design

An important use of cell-type-specific methylation profiles is for the deconvolution of complex mixes of DNA to identify the proportions of contributing cell types. This has the potential to lead to a novel biomarker of ALS: Cell-free DNA (cfDNA) found within plasma is released from dying cells and thus, the quantity of DNA sourced from CNS neurons, and MN in particular, should be proportional to the rate of MN death. Neuronal DNA is not normally seen in the plasma [5], which may be due to a low rate of neuron death or to the blood brain barrier, but brain-derived DNA has been detected in plasma under pathological conditions [21, 22] demonstrating its potential to serve as a biomarker.

**Figure 4:**
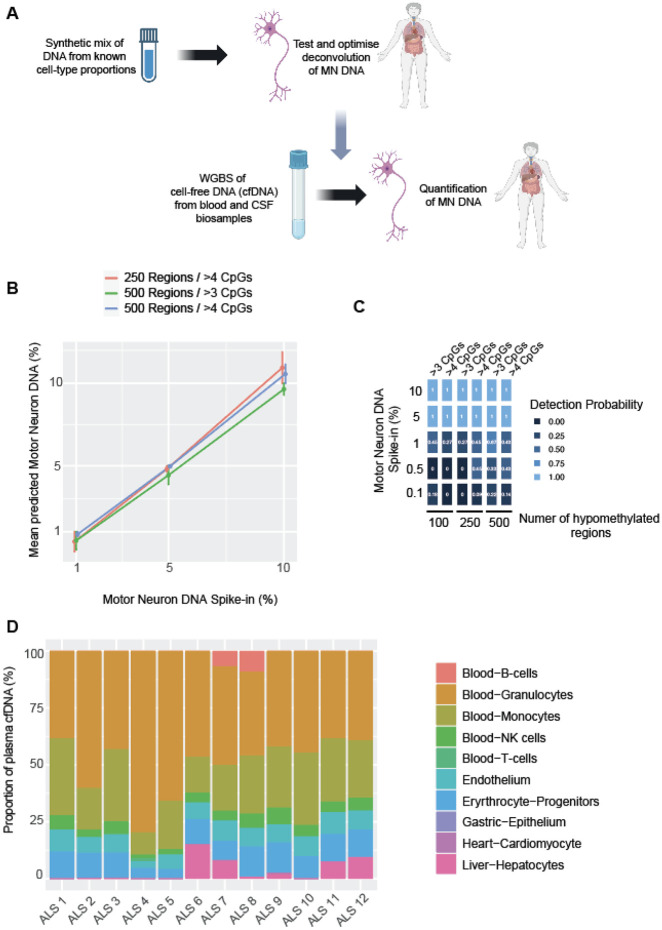
Optimised set of MN-specific hypomethylated genomic regions is not detectable in ALS patient plasma cfDNA. (**A**) We used a synthetic mix of WGBS reads from non-diseased plasma cfDNA together with spike-in reads from iPSC-derived MN to determine the optimum set of MN-specific regions for detection in ALS patient biosamples. **(B)** At spike-ins of 1-10% there is a linear relationship between spike-in and predicted MN DNA concentrations for all sets of MN-specific methylation blocks; p<0.02, adjusted r^2^>0.998, Pearson’s product moment correlation coefficient. (**C**) At spike-ins ≲1% it is possible to detect reads derived from MN-specific regions but the detection probability is <0.5. (**D**) MN-specific DNA is not detectable within ALS patient plasma.

To deconvolute plasma cfDNA we optimised the UXM algorithm [5] for the low coverage (~ 10x) typical of methylation studies of cfDNA; in particular we optimised the choice and configuration of MN-specific methylation blocks. The UXM algorithm was chosen as it makes use of read level methylation data, and has achieved accurate deconvolution of cell types present at proportions as low as 0.1% [5]. Optimisation was performed using synthetic data generated by spiking WGBS data derived from plasma cfDNA of healthy individuals, with sequencing reads derived from human MN at a known proportion between 0.01%-10% (Methods, Fig. 4A). We simulated relatively low coverage (10x) to match coverage in the actual ALS cfDNA samples. We observed a linear correlation between the actual and predicted percentage of spike-in MN DNA with an adjusted r^2^ < 0.9 in all marker sets (Fig. 4B). A configuration of UXM using 500 MN-specific blocks with a minimum of 3 CpGs produced the highest detection probability at 1% spike-in, but 500 blocks with a minimum of 4 CpGs performed better at both 0.5% and 0.1% spike-in (difference in detection probability between 0.1–0.2 at each % spike-in, Fig. 4C). However, we note that at spike-ins of ≤ 0.5%, AUC was poor for all sets of MN marker blocks. The greatest AUC (0.69) at 1% spike-in was achieved with 500 blocks with a minimum of 3 CpGs, in keeping with its higher probability of detection (Supplementary Fig. 1A); this was the configuration taken forward to analyse ALS patient samples.

As seen in [5, 23], deconvolution frequently identified false-positive cell-types within the synthetic mixture (Supplementary Fig. 1B). We used a linear model to examine the effect of coverage and number of marker regions the total number of cell types identified in a sample. Both coverage (p = 0.04) and number of markers (p = 3.7e-4) were significantly negatively correlated with the number of cell types identified, suggesting that increased coverage and using more marker regions per cell-type will reduce the number of cell types falsely identified within a mixture.

### MN-derived DNA is not detectable within plasma cfDNA

When we applied our optimised deconvolution utilising 500 MN-specific methylation blocks with a minimum of 3 CpGs to plasma cfDNA WBGS from n = 12 ALS patients we did not identify MN-derived DNA in any sample (Fig. 4D) suggesting that if MN DNA is present it is below the detectable limit of ~ 1% of plasma cfDNA (Fig. 4B-C).

### Neuronal-derived DNA is detectable in CSF cfDNA

The cerebrospinal fluid (CSF) surrounds the spinal cord and brain, and is encapsulated by the blood brain barrier. It might be expected that CSF cfDNA is enriched in neuronal DNA compared to plasma and so we attempted to fully characterise the contributing cell types within CSF cfDNA (Methods).

No WGBS data was available from ALS patient CSF cfDNA. We analysed four samples of WGBS CSF cfDNA from hydrocephalus patients [24]. Coverage was very low (0.12-0.45x, Supplementary Table 3) due to the low concentration of cfDNA within the spinal cord so samples were merged to improve deconvolution accuracy. We discovered that neuronal and oligodendrocyte DNA comprised 13% and 14% of the total cfDNA with the remainder largely composed of a mix of blood, epithelial, and adipocyte cell types (Supplementary Fig. 1); MN-derived DNA was not detectable in any sample. The contribution of adipocytes may in part reflect the lumbar puncture procedure used to collect CSF as DNA. The lack of a number of CNS-specific cell-types such as microglia within the reference leads to a possible assignment error which is impossible to quantify, and is likely responsible for the small proportion of epithelial and pancreatic cell types identified.

### The theoretical maximum proportion of MN-derived DNA within plasma cfDNA is very low

**Figure 5:**
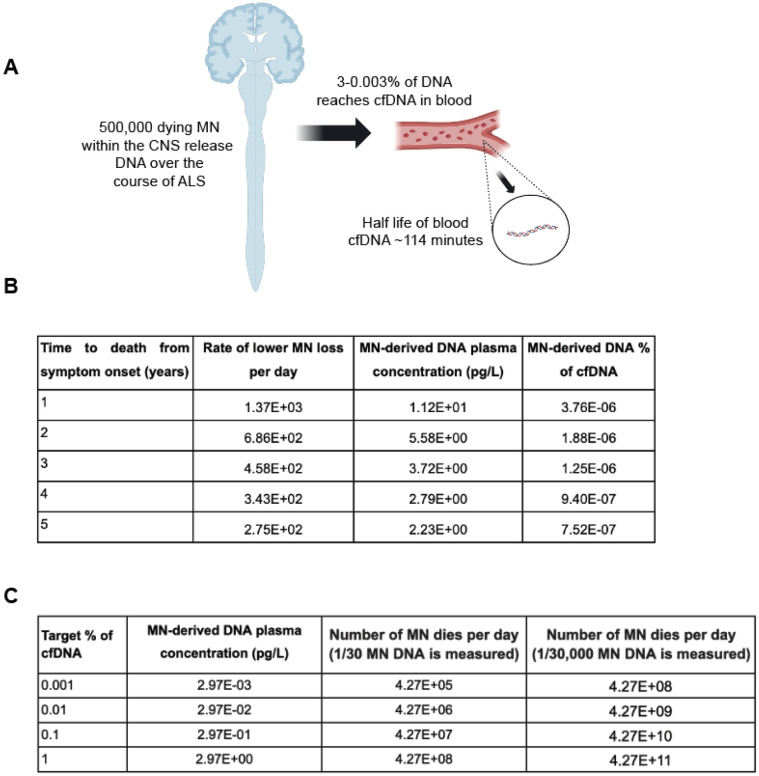
The theoretical maximum proportion of MN-derived DNA within plasma cfDNA is very low. (**A**) We can estimate the proportion of plasma cfDNA derived from MN based on the number of MN dying, the proportion of released DNA which reaches plasma cfDNA and the half-life of cfDNA. (**B**) For different disease durations between one and five years we estimate the proportion of plasma cfDNA derived from MN; and (**C**) we estimate the rate of MN-death necessary to achieve a given concentration within plasma cfDNA.

We did not detect MN DNA in any ALS patient sample suggesting that if MN DNA is present it is below ~ 1% of plasma cfDNA. We questioned if this was a detection deficiency or whether there might be insufficient MN DNA for detection. To address this we modelled the theoretical maximum proportion of MN DNA that might be expected within plasma cfDNA (Fig. 5A).

Recent work [25] has estimated the effect of cellular turnover on the proportion of DNA derived from different cell-types detectable within plasma cfDNA. The proportion of DNA released from dying cells that reaches cfDNA varies dramatically, from 3% of released DNA for megakaryocytes and endothelial cells, to 0.003% for erythrocyte progenitors. Although there are > 86 billion neurons in the human CNS [26], lower MN are a rare subtype of neurons, and previous work has estimated that there may be < 500,000 in total [27]. Assuming optimum availability then 3% of released MN DNA will be detectable within plasma cfDNA. If we assume all lower MN die over the course of disease, we can estimate the theoretical maximum proportion of MN DNA as a part of total plasma cfDNA as a function of the rate of disease progression (Methods, Fig. 5B). From this we can calculate that even for the fastest theoretical disease progression rate, the plasma concentration of MN DNA would be several orders of magnitude smaller than our threshold for detection, primarily because of the small number of MN relative to other cell types. We have assumed a half life for cfDNA of 114 minutes [28]. In our simulation experiments we achieved a detection probability greater than chance only when the proportion of cfDNA attributed to MN was > 1% (Fig. 4B-C) which determined the threshold for theoretical detection.

We sought to estimate what rate of MN death would be required to produce a detectable concentration within cfDNA. Using the proportion of DNA from cellular turnover detectable as cfDNA in the plasma from endothelial cell and erythroblasts as maximum and minimum estimates, we show that even if all lower MN died within 24 hours, their contribution to cfDNA would still be below the limit of detection for WGBS (Fig. 5C). We consider this estimate of wider use to the field as it predicts whether a detectable quantity of cfDNA will be present from a known rate of cell death.

## Methods

### Tissue culture and development of iPSC-derived MN

Tissue culture of iPSCs and the derivation of pure MN cultures via small molecules is described elsewhere [10].

### Whole genome bisulfite sequencing (WGBS) of DNA derived from iPSC-derived MN

We generated WGBS libraries following the Whole-Genome Bisulfite Sequencing Data Standards and Processing Pipeline (https://www.encodeproject.org/data-standards/wgbs/). In brief, genomic DNA was extracted from ~ 50,000 cells per technical replicate before shearing and bisulfite treatment. Libraries were amplified by PCR and purified. Library concentrations were measured (Qubit). WGBS libraries were paired-end sequenced on a NovaSeq 6000 system (Illumina) with target 30X coverage Raw data were processed with the ENCODE 4 pipeline for WGBS according to ENCODE 4 standards. Files are available at encodeproject.org with the following accession numbers: ENCSR734EFX, ENCSR509LMK, ENCSR978LOX.

Paired-end FASTQ files were mapped to the human (hg38), lambda, pUC19 and viral genomes using bwa-meth (v.0.2.0) then converted to BAM files using SAMtools (v.1.9)52. Duplicated reads were marked by Sambamba (v.0.6.5) with parameters ‘-I 1 -t 16 --sort-buffer-size 16000 --overflow-list-size 10000000’ [29]. Reads with low mapping quality, duplicated or not mapped in a proper pair were excluded using SAMtools view with parameters ‘-F 1796 -q 10’. Reads were stripped from nonCpG nucleotides and converted to PAT files using wgbstools (v.0.2.0, downloaded from Github github.com/nloyfer/wgbs_tools in September 2022), command *wgbstools bam2pat* --*genome hg38*. Methylation across the MN samples was examined using a PCA plot, and technical replicates were found to have low heterogeneity. Technical replicates were then merged to allow inclusion in the wgbstools pipeline.

### Genome segmentation into methylation blocks

Using all three of our samples and all 205 samples from a methylation atlas we segmented the genome into 1,630,133 blocks of 4 or more CpGs using the wgbstools command ‘wgbstools segment --min_cpg 4 --max_bp 5000’. PAT and BETA files for all 207 available samples mapped to GRCh38 were downloaded from GEO (accession number GSE186458) [5] on the 20th of September 2022. As per the original publication we excluded two cardiomyocyte samples due to low coverage. We also segmented the genome into 1,938,130 blocks of 3 CpGs were identified using the wgbstools command wgbstools segment --min_cpg 3 --max_bp 5000; these blocks of 3 CpGs were used only for marker selection.

### Unsupervised clustering of DNA methylation profiles

Average methylation per block (of at least 4 CpGs in size) for each sample was extracted using the wgbstools command ‘beta_to_table’, replacing blocks with less than 10x coverage in a sample with ‘NA’. We then selected the top 1% of blocks by variance, excluding blocks with any ‘NA’ values across all samples, and used these for clustering. Unsupervised clustering was performed using Python version 3.10.8, Dask version 2023.9.2, SciPy 1.9.1, options method='average', metric='cityblock', optimal_ordering = True.

### Derivation of MN-specific hypomethylated genomic regions

We applied the wgbstools command ‘find_markers’ together with all 205 samples used for segmentation. Default parameters were used to remove low coverage regions, samples with a read depth of less than 5 in a segment had the value set to NA, and segments with greater than 1 in 3 NA values in either the target or background cell type were removed. Regions were considered MN-specific if there was a difference of at least 0.3 between the mean motor neuron methylation and mean of all other samples’ methylation within that block, and the p value of a t-test was equal to or below 0.05.

### Identification of genes linked to MN-specific hypomethylated genomic regions

We implemented the ABC model [13] following the guidelines provided at https://github.com/broadinstitute/ABC-Enhancer-Gene-Prediction. First, we called peaks for the ATAC-seq profiling using MACS2, and then identified the candidate enhancer elements using “makeCandidateRegions.py” with parameters peakExtendFromSummit = 250 and nStrongestPeaks = 150000. The black-listed regions generated by the ENCODE 4 (https://www.encodeproject.org/) were used for removing enhancers overlapping regions with anomalous sequencing reads. Second, we applied “run.neighborhoods.py” to quantify the enhancer activities by counting ATAC-seq and H3K27ac ChIP-seq reads in candidate enhancer regions. RNA-seq profiling of iPSC-derived MNs was also provided to inform expressed genes. Quantile normalisation was applied using K562 epigenetic data as the reference. At last, using “predict.py” we computed the ABC scores by combining the enhancer activities (calculated by the second step) with the Hi-C profiling. Hi-C data was fit to the power-law model. The default threshold 0.02 was used to define valid E-P links.

### Transcriptome analysis

For AnswerALS data, gene expression profiling of iPSC-derived MNs and phenotype data were obtained for 245 ALS patients and 45 neurologically normal controls (https://www.answerals.org/). Gene expression was normalised by the The trimmed mean of M-values normalisation method (TMM). We used a negative binomial test to determine genes differentially expressed between ALS patients and controls. Significance testing was performed for all genes expressed in MN (n = 22,976) defined as count above zero in more than half of samples; in addition we excluded the bottom 25% of genes based on mean count across all samples.

### Generation of synthetics mixes of MN-derived DNA together with plasma cfDNA

WGBS of plasma cfDNA samples produced by Caggiano C. et al. [12] were downloaded from https://www.ncbi.nlm.nih.gov/geo/query/acc.cgi?acc=GSE164600 in February 2023, including 12 ALS patients and 12 healthy volunteers. Raw FastQ files were trimmed with Trim Galore version 6.7 using the options ‘trim_galore --paired -clip_R1 4 --clip_R2 4 --three_prime_clip_R1 12 --three_prime_clip_R2 12’ and then aligned to GRCh38 using the bowtie 2 aligner in Bismark version 22.3. Duplicate reads were removed with Bismark and Samtools version 1.16.1 was used to remove reads with a MAPQ score below 10. BAM files were then converted to PAT and BETA files using wgbstools.

Using wgbstools command ‘mix_pat’, synthetic mixes of MN sample PGP_M_55_iPSC (Supplementary Table 1) or cerebral neuron sample Cortex-Neuron-Z0000042F [5] and the either the 12 plasma cfDNA samples from healthy volunteers, or the 4 CSF cfDNA samples from hydrocephalus patients were created. By down- or up-sampling the cfDNA and neuronal reads, spike-ins were made at 0–10%, and coverage was varied from 2.5-30x.

### Deconvolution of plasma cfDNA and optimisation of a deconvolution algorithm

We derived uniquely hypomethylated regions for each cell-type to use for deconvolution. In this process we excluded the two samples used for spike-in to prevent overfitting. Segmentation was repeated as before to derive two sets of regions, one with a minimum length of 3 CpGs and one with a minimum length of 4 CpGs. For both sets of regions cell type specific marker regions were found using wgbstools ‘find_markers’ with a minimum difference between target and background means of 0.3 and a t-test p-value equal to or below 0.05. To derive different numbers of marker regions, for each cell-type the marker regions were ordered by the difference between the 75th-centile in the target group and the 2.5th centile in the background and then 25, 50, 100, 250, 300, 400, or 500 marker regions were selected. Marker regions for all cell types were then used to create an atlas of the fragment based methylation for each region across all cell types using the UXM tool downloaded from https://github.com/nloyfer/UXM_deconv on the 31st of January 2023. We then used UXM to deconvolve the synthetic mixes, producing estimated cell type contributions for each mix. These were then analysed using R version 4.3.1 (2023-06-16). To optimise region selection we tested using smaller or larger regions, and more or less regions per cell-type in order to maximise the probability of detection of spiked-in DNA, and minimise the normalised root mean squared error (RMSE).

### Deconvolution of CSF cfDNA

WGBS of CSF cfDNA samples [30] were downloaded from https://www.ncbi.nlm.nih.gov/geo/query/acc.cgi?acc=GSE142241 in April 2023, including four hydrocephalus patients. Reads were trimmed with trim-galore version 6.7 using the paired option and default settings. Due to low mapping efficiency of the reads we followed the ‘Dirty Harry’ protocol described by the creators of the Bismark software. Reads were first aligned as paired end reads using the bowtie aligner within Bismark. Unmapped R1 reads were then aligned in directional mode, and R2 reads were then aligned in pbat mode before combining them into a single file. Duplicate reads were then removed with Bismark, then Samtools version 1.16.1 was used to remove reads with a MAPQ score below 10 before converting them into PAT and Beta files using wgbstools.

### Theoretical estimate of the maximum of MN-derived DNA within plasma cfDNA

The concentration of cfDNA produced from cell death is given by the standard pharmacokinetic equation for concentration produced by a drug infusion at a constant rate.


$$C=d(k_{0}*t_{1∕2})\;∕\;(\;\ln(2)\;*\;\mathrm{Vd}\;)$$

Where C is the concentration in the plasma, $k_{0}$ is the infusion rate, $t_{1∕2}$ is the half life, Vd is the volume of distribution, and d is the proportion of DNA from cell death present in the plasma. We were able to calculate the theoretical maximum concentration of MN DNA within plasma cfDNA as a function of the time period over which the DNA was released i.e. disease duration by making reasonable assumptions for each of these values. Using the values given for a 70kg 20–25 year old man as has historically been used as standard, the volume of plasma is 3.0L [31]. In the absence of a ground-truth for the proportion of DNA released from dying MN that reaches plasma cfDNA, we used observed maximum and minimum proportions for other cell-types: from 3% for megakaryocytes and endothelial cells to 0.003% for erythrocyte progenitors [25]. Infusion rate is given by the rate of cell death, and converted to weight of DNA using the conversion 1 diploid genome = 6.46pg [32]. The total number of lower MN has been estimated at ~ 500,000 [27] and we estimate a constant rate of loss over the disease course based on the observation that neurofilament levels, a biomarker of neuronal death, rise prior to disease onset then reach a stable concentration that is proportional to speed of progression [33]. The half life of plasma cfDNA has been measured using a variety of means, including the decrease in foetal cfDNA following pregnancy, the decrease in tumour cfDNA following surgery, and the increase and decrease in cfDNA following exercise [34]. A key point is to distinguish between the distribution half life and steady state half life. As shown by experiments with radiolabeled double stranded DNA [35], following an infusion DNA is taken up by soft tissues causing its concentration in the plasma to decrease rapidly until an equilibrium is reached with equal movement of DNA between the soft tissues and plasma. Following this the concentration of DNA will reach a steady state where its concentration is determined by the infusion rate and the steady state half life. We use 114 minutes as our estimate for the steady state half life as this is based on the fall in circulating tumour DNA following complete resection of the tumour [36]. cfDNA from the tumour would have reached a steady state prior to the surgery and its decrease from the surgery would be in line with the steady state half life. When estimating the proportion of cfDNA we use the concentration of 297pg/ul as the expected concentration of plasma cfDNA as this was the average concentration in controls age and sex matched to ALS patients [12].

## Discussion

ALS is currently an incurable and invariably fatal neurodegenerative disease [37]. Biomarkers are crucial for translational medicine and the recent development of serum NfL as a biomarker for ALS [1] has been key to the development of new treatments [38]. However, a key deficiency of NfL measurement is that it is not specific to MN [3], the primary degenerating cell in ALS. We and others have hypothesised that detection of cell-specific methylation of DNA within plasma cfDNA might provide an alternative and more specific biomarker for ALS. Here we show theoretically and experimentally that this goal is potentially not achievable using WGBS of plasma cfNDA, at least under the experimental conditions we encountered. Alternative approaches are needed which may include alternative biofluids or detection methods.

We have developed a MN-specific set of hypomethylated genomic regions using WGBS in iPSC-derived MN from neurologically normal individuals, together with an atlas of tissue-specific methylation [5]. We demonstrate that these regions are associated with genes which are key to MN function but not significantly enriched with ALS genetic risk. Our regions are likely to be useful for future works aiming to detect DNA derived from MN using different detection methods.

Our simulations and our measurements suggest that the sensitivity of WGBS is limited to 1% of plasma cfDNA which is significantly greater than the theoretical maximum proportion of plasma cfDNA derived from rapidly degenerating MN, which we determine to be 1.6*10^−5^%. This is due to the relatively small number of MN compared to the ongoing turnover of other cell-types. It is not inconceivable that MN-derived DNA could be detected at this level but targeted amplification together with more sensitive detection will be necessary.

An important limitation to our work, and the majority of deconvolution algorithms, is that they assume the sequenced DNA fragments are randomly distributed across the genome, which is not correct. It is known that the formation of cell-free DNA from genomic DNA leads to preferential preservation of nucleosome-bound DNA, so cell-free DNA from different cell types or tissues produces fragmentation patterns with greater depth at sites bound to nucleosomes [39]. Enrichment of MN-specific methylation blocks used for detection with nucleosome-bound genomic regions could potentially improve the performance of detection.

It is possible that use of an alternative biofluid might enable detection of MN-specific DNA. CSF is the obvious choice given that, unlike blood, it is not separated from MN by the blood brain barrier (BBB). However, the extremely low concentration of cfDNA in CSF – 0.4ng/mL versus 7.7ng/mL in plasma [40] – may again be prohibitive. Our preliminary analysis suggests that neuronal but not MN-derived DNA is detectable within CSF cfDNA via WGBS, but this did not include sequencing data from ALS patients.

Our study has contributed WGBS data from iPSC-derived MN (encodeproject.org, Methods) and the identification of MN-specific hypomethylated genomic regions. We have not achieved a new biomarker for ALS but we have delineated the challenge for this approach through both theoretical calculations and experimental measurements. We have shown that WGBS of cfDNA derived from plasma is not likely to lead to a new biomarker for ALS and that future research should focus on developing our MN-specific regions with a more sensitive detection method.
